# Fasudil Attenuates Concanavalin A-Induced Autoimmune Hepatitis and Is Associated with Suppression of RhoA/ROCK and TLR4/NF-κB Signaling and Modulation of Immune Responses

**DOI:** 10.3390/ph19081237

**Published:** 2026-08-06

**Authors:** Reem A. Alzoubi, Ahmed M. Awad, Marwa E. Abdelmageed

**Affiliations:** 1 Department of Pharmacology and Toxicology, Faculty of Pharmacy, Mansoura University, Mansoura 35516, Egypt; reemalaa664@gmail.com (R.A.A.); marwaelsayed90@mans.edu.eg (M.E.A.); 2 Department of Pharmacology and Toxicology, Faculty of Health Science Technology, Mansoura National University, Mansoura 7731168, Egypt

**Keywords:** autoimmune hepatitis, fasudil, ROCK, inflammation, cytokines, apoptosis

## Abstract

**Background/Objectives**: Autoimmune hepatitis (AIH) is a liver injury characterized by the dysregulation of immune responses. Current options for AIH have limitations that highlight the urgent need to investigate alternative or adjunct therapeutic strategies for its management. We aimed to explore the protective effects of fasudil against concanavalin A (Con A)-induced AIH in mice. Con A resulted in significant hepatic damage, evidenced by dysregulation in liver function biomarkers, marked inflammatory cell infiltration, and hepatocellular degeneration. **Methods:** The pretreatment of mice with fasudil (10 and 25 mg/kg, intraperitoneally) for 7 days resulted in a significant ameliorative effect on these alterations in a dose-dependent manner. Fasudil treatment was associated with suppression of the RhoA/Rho-associated coiled-coil-containing protein kinase (ROCK) pathway and concomitant downregulation of toll-like receptor 4 (TLR4)/Nuclear factor-κB (NF-κB). **Results**: These changes were accompanied by reduced macrophage activation and the reduction of pro-inflammatory cytokines, as evidenced by decreased nitric oxide synthase (iNOS), tumor necrosis factor alpha (TNF-α), interleukin-6 (IL-6), interferon gamma (INF-γ), and interleukin-17A (IL-17A). Moreover, it significantly limited the cluster of differentiation (CD)4^+^ and CD8^+^ T cell infiltration, thereby modulating adaptive immune amplification. **Conclusions:** Accordingly, fasudil restored redox homeostasis and mitigated hepatocellular death by rebalancing apoptotic regulators, normalizing the expression of B cell lymphoma 2 (BCL-2) while reducing the expression of BCL-2-associated X protein (BAX) and caspase-3 activation. Subsequently, fasudil improved liver function profiles and preserved hepatic architecture. Our results demonstrate that fasudil has potent immunomodulatory, anti-inflammatory, antioxidant, and anti-apoptotic effects in AIH. Its protective effects are associated with the suppression of RhoA/ROCK and TLR4/NF-κB signaling, supporting further investigation into the contribution of these pathways to the therapeutic actions of fasudil in AIH.

## 1. Introduction

Liver injuries represent the most common abdominal injury. Autoimmune hepatitis (AIH) is a progressive liver inflammation mediated by an autoimmune response [1]. It has an underestimated prevalence and unsatisfactory complete remission therapeutic output [2]. The prevalence and clinical manifestations of AIH can be linked to various factors, including environmental influences, genetic factors, medications, and ethnic background, which can lead to complications such as liver cirrhosis and hepatocellular carcinoma [3].

Among the guidelines for AIH management, corticosteroids and azathioprine emerge as first-line options [4,5]. Although they are effective in many patients, they have some limitations, including relapse after their withdrawal [6], incomplete response or failure in some patients [7], and adverse effects such as cytopenia, arthralgias, and gastrointestinal toxicity [8,9]. This highlights the urgent need to investigate alternative or adjunct therapeutic options for AIH.

There is crosstalk between the disease and immune cell activation, which, if left untreated, may progress to fibrosis and cirrhosis [10]. Among these immune cells, CD4^+^ T cells play a central role in adaptive immune response [11]. CD4^+^ T cell abnormalities can result in severe autoimmune diseases, and prominent infiltration is observed in the livers of AIH patients [12].

Cytokine-induced inflammation is tightly linked with intracellular signaling networks, among which Rho-associated coiled-coil-containing protein kinases (ROCKs) have a central role [13]. ROCK2, among the two ROCK isoforms, has emerged as a key regulator of T cell differentiation, immune cell activation, macrophage function, and pro-inflammatory cytokine production. Accordingly, it is particularly relevant to autoimmune and inflammatory diseases [14]. Consequently, the present study focuses on ROCK2 as the predominant ROCK isoform implicated in immune-mediated inflammation. There is also a crosstalk between it and the TLR4/NF-κB signaling pathway [15]. This highlights its relevance in immune-mediated diseases.

Although concanavalin A (Con A)-induced hepatitis is primarily initiated by T cell activation, accumulating evidence indicates that innate immune signaling substantially amplifies liver injury. Hepatocyte damage induced by activated T cells promotes the release of damage-associated molecular patterns (DAMPs), which activate Kupffer cells and infiltrate macrophages through Toll-like receptor 4 (TLR4). Subsequent activation of nuclear factor-κB (NF-κB) enhances the production of pro-inflammatory mediators, including tumor necrosis factor-α (TNF-α), interleukin-6 (IL-6), and inducible nitric oxide synthase (iNOS), thereby amplifying hepatic inflammation and promoting the further recruitment and activation of immune cells [16,17,18]. Thus, although T cell activation initiates Con A-induced hepatitis, the TLR4/NF-κB pathway functions as an important amplifier of the inflammatory cascade and represents a potential therapeutic target.

There are various animal models that have been investigated to mimic AIH pathogenesis [19]. However, it was reported that AIH induced by Con A is the most relevant animal model [20]. Notably, Con A is a potent stimulator of T cells that have a central role in AIH [21]. This stimulation by Con A results in the overproduction of a wide array of inflammatory mediators, which results in a phenomenon called a “cytokine storm.” Accordingly, this cytokine storm sustains and amplifies immunostimulant processes [22]. Con A has another advantage as a model for AIH because it affects the liver without adversely affecting the heart or lungs [18]. Altogether, Con A as an AIH model is considered one of the most relevant experimental models.

Fasudil is a potent ROCK inhibitor [23] that has been approved in both Japan and China for cerebral vasospasm after subarachnoid hemorrhage [24]. Fasudil-induced ROCK inhibition was found to provide benefits for neurodegenerative diseases [25]. Moreover, it has shown promising results in various preclinical studies of cardiovascular diseases [26,27]. We hypothesize that inhibition of the RhoA/ROCK2 pathway by the ROCK inhibitor fasudil could ameliorate Con A-induced AIH by modulating T-lymphocyte activation and the TLR4/NF-κB signaling pathway. To the best of our knowledge, this is the first study to investigate the therapeutic potential of fasudil in AIH. Evaluating fasudil in this setting may provide new insights into the immunomodulatory potential of ROCK2 inhibition in autoimmune liver inflammation.

## 2. Results

At the outset of this study, the fasudil 25 mg/kg CTRL group was first included in the trial and checked for histopathological alterations and general and oxidative stress biomarkers. The results demonstrate that fasudil alone did not produce any detectable effects on these parameters. Therefore, the following analyses were conducted on the primary experimental arms (Control, Con A, fasudil 10 + Con, and fasudil 25 + Con A) to highlight the molecular pathways involved in facudil’s protective effects against Con A-induced AIH in mice.

### 2.1. Effect of Fasudil on Histopathological Changes Induced by Con A

**Figure 1:**
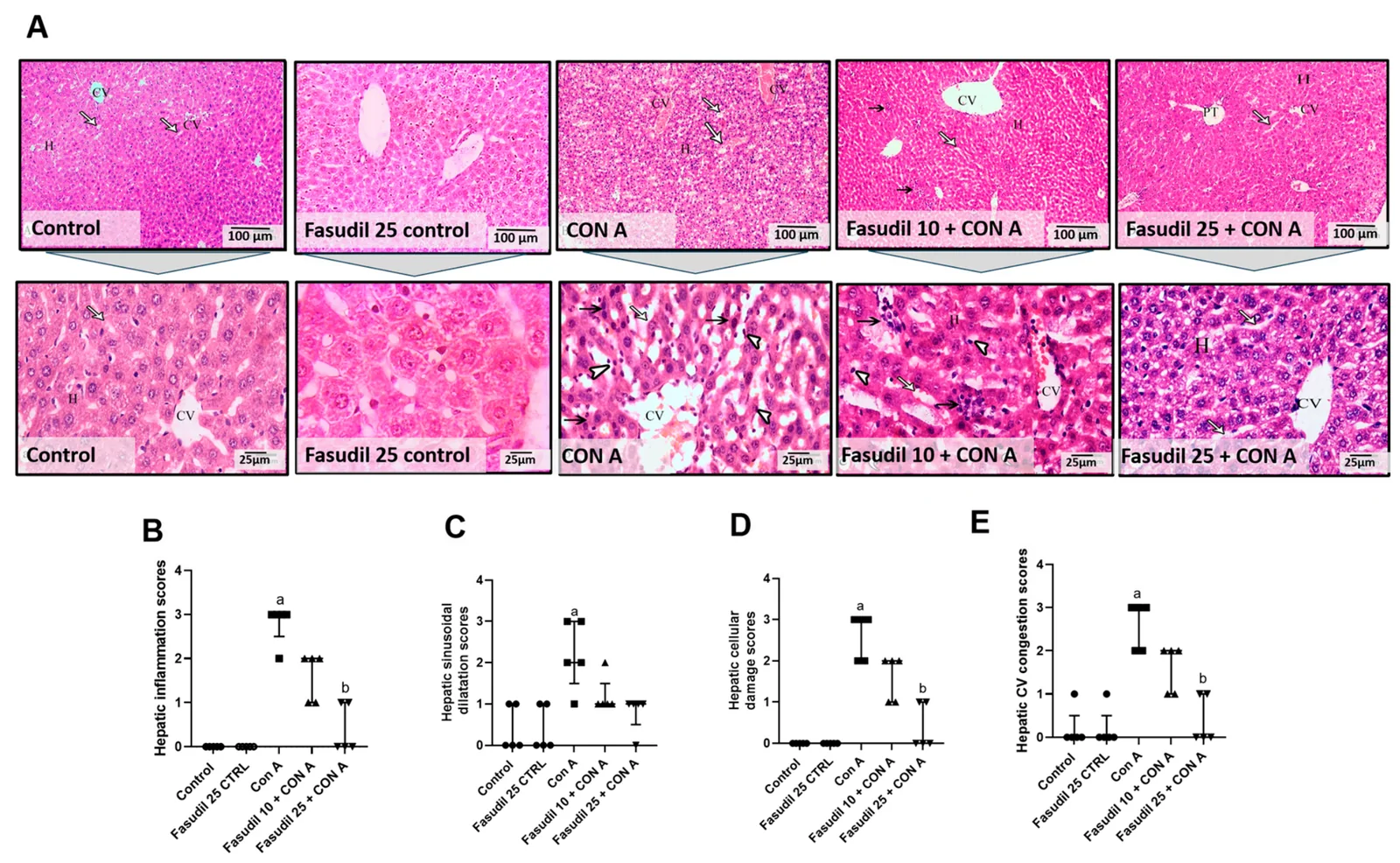
Effect of fasudil on Con A-induced changes in hepatic histopathology. Image magnification = 100×; inset = 400×. (**A**) Representative histological images of mice liver, (**B**) scatter dot graph showing the score for inflammation, (**C**) scatter dot graph showing the score for sinusoidal dilation, (**D**) scatter dot graph showing the score for cellular damage, the (**E**) scatter dot graph showing the score for central vein (CV) congestion. Arrows: arrows indicate blood sinusoids; curved arrows indicate hepatocyte nuclei; arrowheads indicate activated Kupffer cells; black arrows indicate shrunken hepatocytes with dark acidophilic cytoplasm and inflammatory cell infiltration, as indicated in the corresponding panel. Data were statistically analyzed using Kruskal Walis test followed by Dunn multiple comparisons test. Data are expressed as median ± interquartile range, *n* = 5. ^a,b^ *p* < 0.05 was considered significantly different from the control and Con A groups, respectively.

As shown in Figure 1A, H&E-stained micrographs of mouse liver tissue sections from the control group showed a normal liver structure with polyhedral hepatocytes arranged in strands, alternating with blood sinusoids that formed a network around the central vein. Meanwhile, the Con A-treated group showed central vein congestion, sinusoidal dilation, increased Kupffer cell activity, cellular damage, and inflammatory cell infiltration. On the other hand, the Fasudil 10 + Con A-treated group showed moderate central vein congestion, moderate sinusoidal dilatation, inflammatory cell infiltration, and minimal Kupffer cell activation among hepatocytes. Some hepatocytes were shrunken with dark acidophilic cytoplasm, whereas the Fasudil 25 + Con A-treated group showed a normal central vein separated by blood sinusoids with no inflammatory cell infiltration. Treatment with fasudil in both the Fasudil (10 and 25 mg/kg) groups significantly attenuated liver inflammation compared to the Con A group.

Scoring of hepatic inflammation (Figure 1B), sinusoidal dilation (Figure 1C), cellular damage (Figure 1D), and CV congestion (Figure 1E) demonstrated a significant increase in Con A-injection mice compared to the control group. Treatment with fasudil (10 and 25 mg/kg) led to a decrease in all previously recorded scores in a dose-dependent pattern.

### 2.2. Effect of Fasudil on Liver Function Biomarkers in Mice Induced by Con A

**Figure 2:**
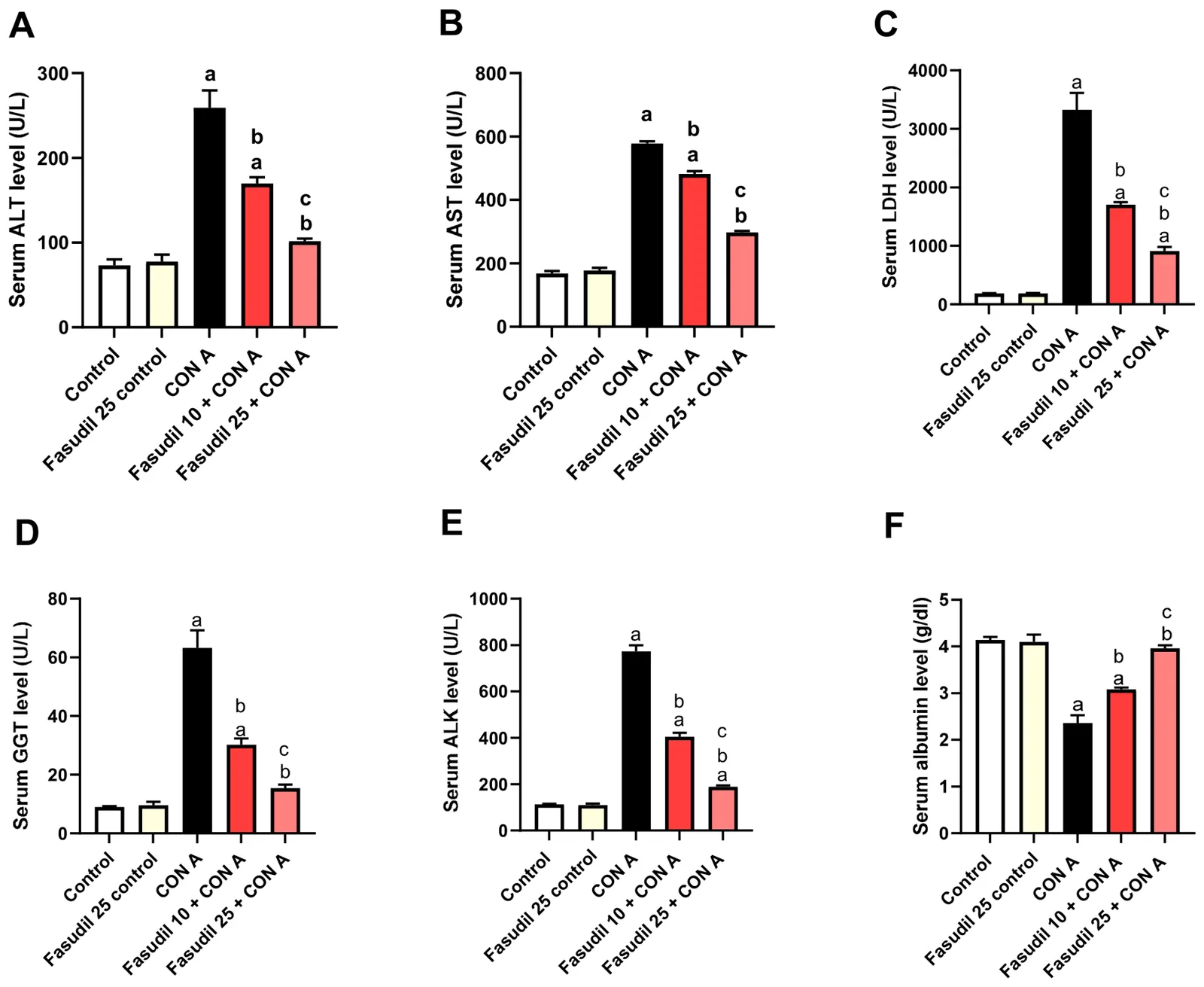
Fasudil attenuates concanavalin A (Con A)-induced elevations of liver function biomarkers. (**A**) Alanine aminotransaminase (ALT), (**B**) aspartate aminotransferase (AST), (**C**) lactate dehydrogenase (LDH), (**D**) Gamma-Glutamyl Transferase (GGT), (**E**) Alkaline Phosphatase (ALK), and (**F**) Albumin (ALB). Data are expressed as mean ± SEM, *n* = 4. One-way analysis of variance (ANOVA) followed by Tukey–Kramer multiple comparisons post hoc test. *p* ≤ 0.05 was considered statistically significant. ^a,b,c^, *p* ≤ 0.05 was considered significantly different relative to the control, Con A, or Fasudil 10 + Con A groups, respectively.

Con A markedly disrupted liver function, as shown by dramatic elevations in ALT, AST, LDH, GGT, and ALK levels (Figure 2A–E), while simultaneously lowering serum ALB levels (Figure 2F). Pretreatment with fasudil (10 and 25 mg/kg) significantly improved the aforementioned parameters compared to the Con A group. Notably, the higher dose (25 mg/kg) showed a significantly greater hepatoprotective effect than the lower dose (10 mg/kg).

### 2.3. Effect of Fasudil on Hepatic Oxidative Stress and Antioxidant Biomarkers Induced by Con A

**Figure 3:**
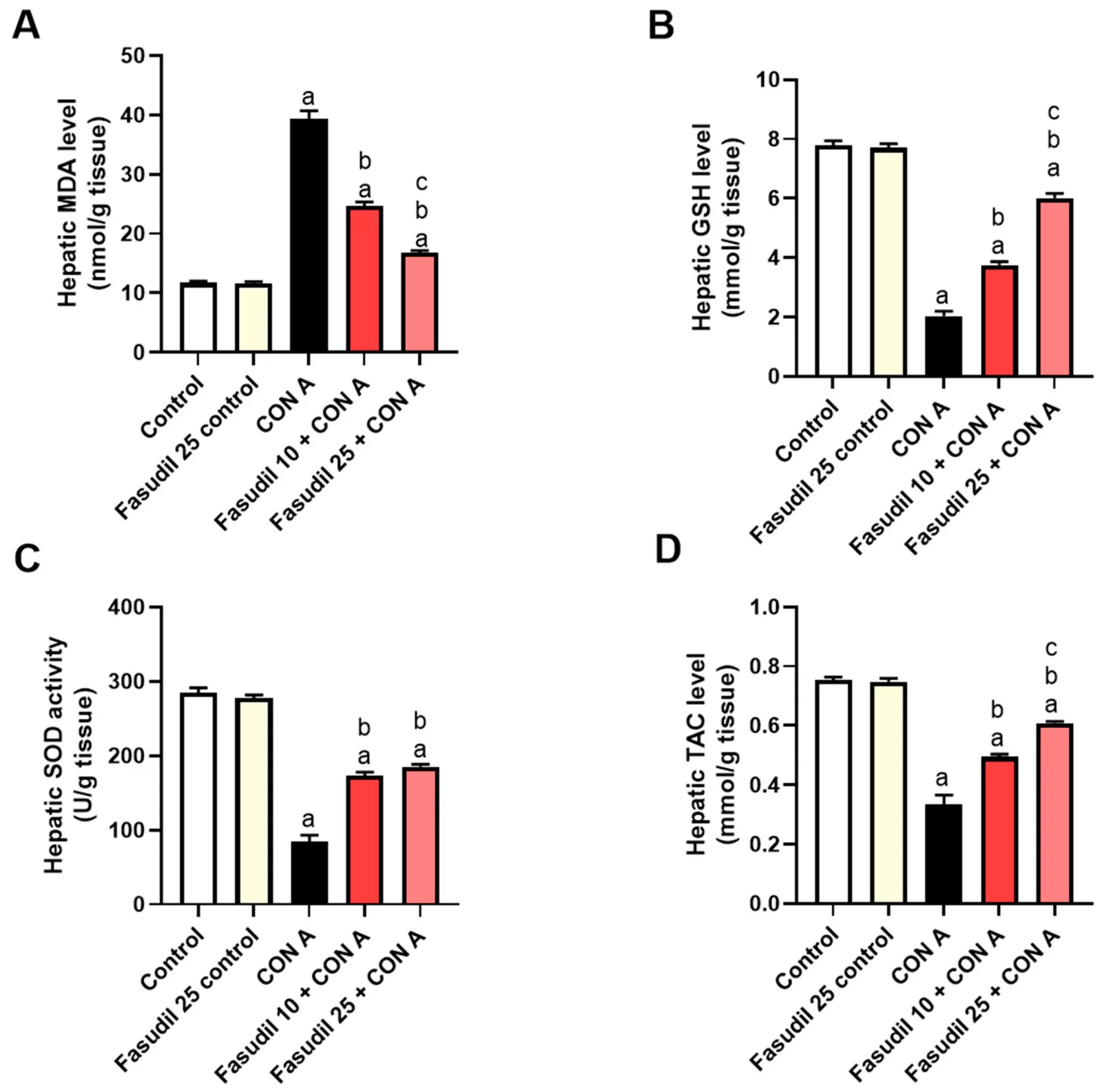
Fasudil protects hepatic tissue against concanavalin A (Con A)-mediated oxidative stress. (**A**) Malondialdehyde (MDA), (**B**) Glutathione (GSH), (**C**) superoxide dismutase (SOD), and (**D**) total antioxidant capacity (TAC). These measurements were performed using liver tissue homogenates. Data are expressed as mean ± SEM, *n* = 5. One-way analysis of variance (ANOVA) followed by Tukey–Kramer multiple comparisons post hoc test. *p* ≤ 0.05 was considered statistically significant. ^a,b,c^, *p* ≤ 0.05 was considered significantly different relative to the control, Con A, or Fasudil 10 + Con A groups, respectively.

Another deriver of Con A-induced AIH is oxidative stress [28], which alters gene expression and promotes apoptosis, necrosis, and fibrosis [29]. The RhoA/ROCK pathway has been reported to play a role in oxidative stress in multiple pathological conditions [30,31]. Consistently, as shown in Figure 3, the Con A group significantly increased hepatic MDA levels (Figure 3A) compared to the control group. On the other hand, Con A resulted in a significant decrease in GSH (Figure 3B), SOD (Figure 3C), and TAC (Figure 3D) compared to the control group. Pretreatment with fasudil (10 and 25 mg/kg) significantly decreased MDA levels and increased SOD activity, GSH levels, and TAC levels compared to the Con A group, with the higher dose (25 mg/kg) having a more significant hepatoprotective effect than the lower dose (10 mg/kg).

### 2.4. Effect of Fasudil on Hepatic RhoA and ROCK2 Induced by Con A

**Figure 4:**
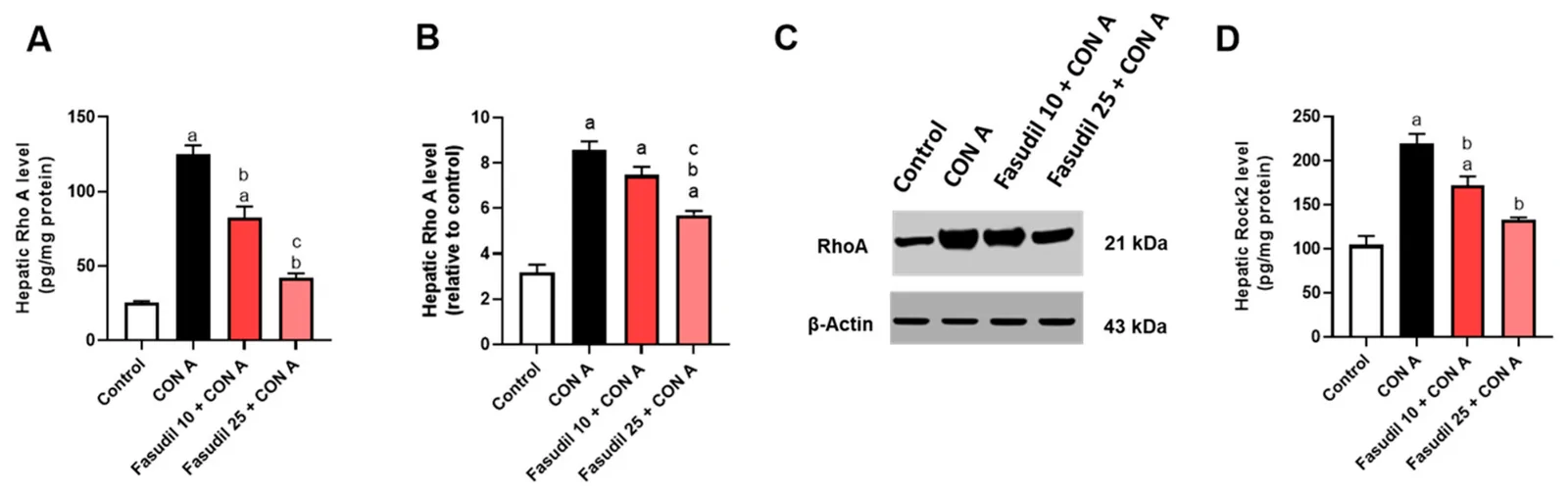
Inhibitory effect of Fasudil on concanavalin A (Con A)-induced RhoA/ROCK2 signaling: (**A**) Ras homolog gene family member (Rho A) levels (obtained by ELISA), (**B**) protein expression of Rho A, (**C**) Rho A representative bands (obtained by Western blot), and (**D**) hepatic levels of Rho-associated coiled-coil-containing protein kinase 2 (ROCK2) (obtained by ELISA). These ELISA measurements were performed using liver tissue homogenates. One-way ANOVA followed by Tukey–Kramer multiple comparisons post hoc test. Data are expressed as mean ± SEM, *n* = 4, *p* ≤ 0.05 was considered statistically significant. ^a,b,c^, *p* ≤ 0.05 was considered significantly different relative to the control, Con A, or Fasudil 10 + Con A groups, respectively.

There is a crosstalk between RhoA/ROCK2 signaling and lymphocyte activation, proliferation, and differentiation. Although fasudil inhibits both ROCK1 and ROCK2, the present study focused on ROCK2 because of its well-established role in immune regulation and inflammatory signaling. Consistently, our study shows that inhibition of the hepatic levels of RhoA and ROCK2 help us to study its role and associate the effect of fasudil on AIH with this pathway. Interstingly, as shown in Figure 4, the hepatic RhoA level and protein expression (Figure 4A–C) and the level of ROCK2 (Figure 4D) were significantly increased in the Con A group compared to the control group. Pretreatment with fasudil (10 and 25 mg/kg) significantly decreased these levels compared to the Con A group.

### 2.5. Effect of Fasudil on CD4^+^ and CD8^+^ T Cell Infiltration Induced by Con A

**Figure 5:**
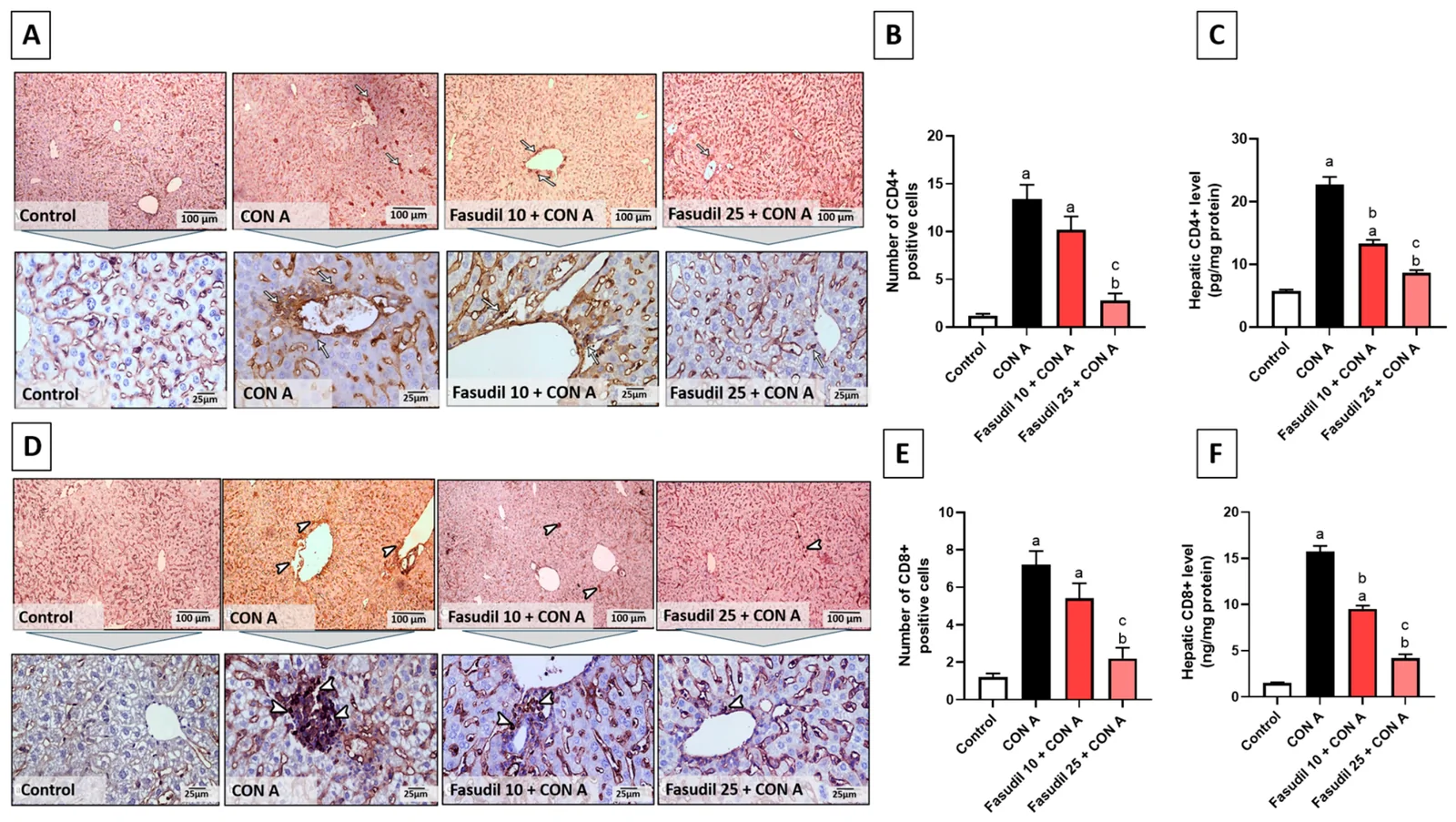
Protective effect of fasudil against hepatic CD4^+^ and CD8^+^ upregulation induced by Con A. (**A**) CD4^+^ immune figure; (**B**) bar graph showing number of CD4^+^ positive cells (obtained by IHC); (**C**) CD4^+^ level (obtained by ELISA); (**D**) CD8^+^ immune figure; (**E**) bar graph showing number of CD4^+^ positive cells (obtained by IHC); (**F**) CD8^+^ level (obtained by ELISA). CD−: cluster of differentiation; Con A: concanavalin A. Arrows indicate CD4^+^ and CD8^+^ T-cell infiltration in the liver tissue. Data are expressed as mean ± SEM, *n* = 4. One-way ANOVA followed by Tukey–Kramer multiple comparisons post hoc test. *p* ≤ 0.05 was considered statistically significant. ^a,b,c^, *p* ≤ 0.05 was considered significantly different relative to the control, Con A, or Fasudil 10 + Con A groups, respectively.

The migration of T lymphocytes across the endothelium was reported to be highly dependent on the RhoA/ROCK signaling pathway, as efficient transmigration requires coordinated contraction of the T cell uropod during cell movement [32]. ROCK activity has also been shown to be enriched within filamentous and tubular pseudopodia of migrating T cells, where it regulates key processes, including adhesion, migration, differentiation, proliferation, and survival. Increases in ROCK activation have been consistently observed in T cells from autoimmune disease mouse models [33,34]. To study the effect of fasudil on T cell infiltration, we performed immunohistochemical evaluation, as represented in Figure 5. Photomicrographs representing immuno-stained CD4^+^ liver sections of the control group revealed no CD4^+^ T cell infiltration; on the other hand, the Con A-injected group revealed marked CD4^+^ T cell infiltration. Meanwhile, the fasudil (10 mg/kg) pretreated group revealed moderate CD4^+^ T cell infiltration, and the fasudil (25 mg/kg) pretreated group revealed minimal CD4^+^ T cell infiltration (Figure 5A). Additionally, photomicrographs representing immuno-stained CD8^+^ liver sections of the control group revealed no CD8^+^ T cell infiltration; on the other hand, the Con A-injected group revealed marked CD8^+^ T cell infiltration. Meanwhile, the fasudil (10 mg/kg) pretreated group revealed moderate CD8^+^ T cell infiltration, and the fasudil (25 mg/kg) pretreated group revealed minimal CD8^+^ T cell infiltration (Figure 5D).

Con A administration upregulated the immune expression of CD4^+^ (Figure 5B) and CD8^+^ (Figure 5E) cell-positive stained areas compared to the control group. Co-treatment with the fasudil (10 and 25 mg/kg) group significantly lowered the immune expression of CD4^+^ and CD8^+^ compared to the Con A group. Notably, fasudil (25 mg/kg) pretreatment was significantly better than fasudil (10 mg/kg). As revealed in Figure 5C,F, the Con A group significantly increased hepatic CD4^+^ and CD8^+^ levels compared to the control group. Pretreatment with fasudil (10 and 25 mg/kg) significantly decreased CD4^+^ and CD8^+^ levels compared to the Con A group.

### 2.6. Effect of Fasudil on INF-γ, TNF-α, IL-6 and IL-17A Induced by Con A

**Figure 6:**
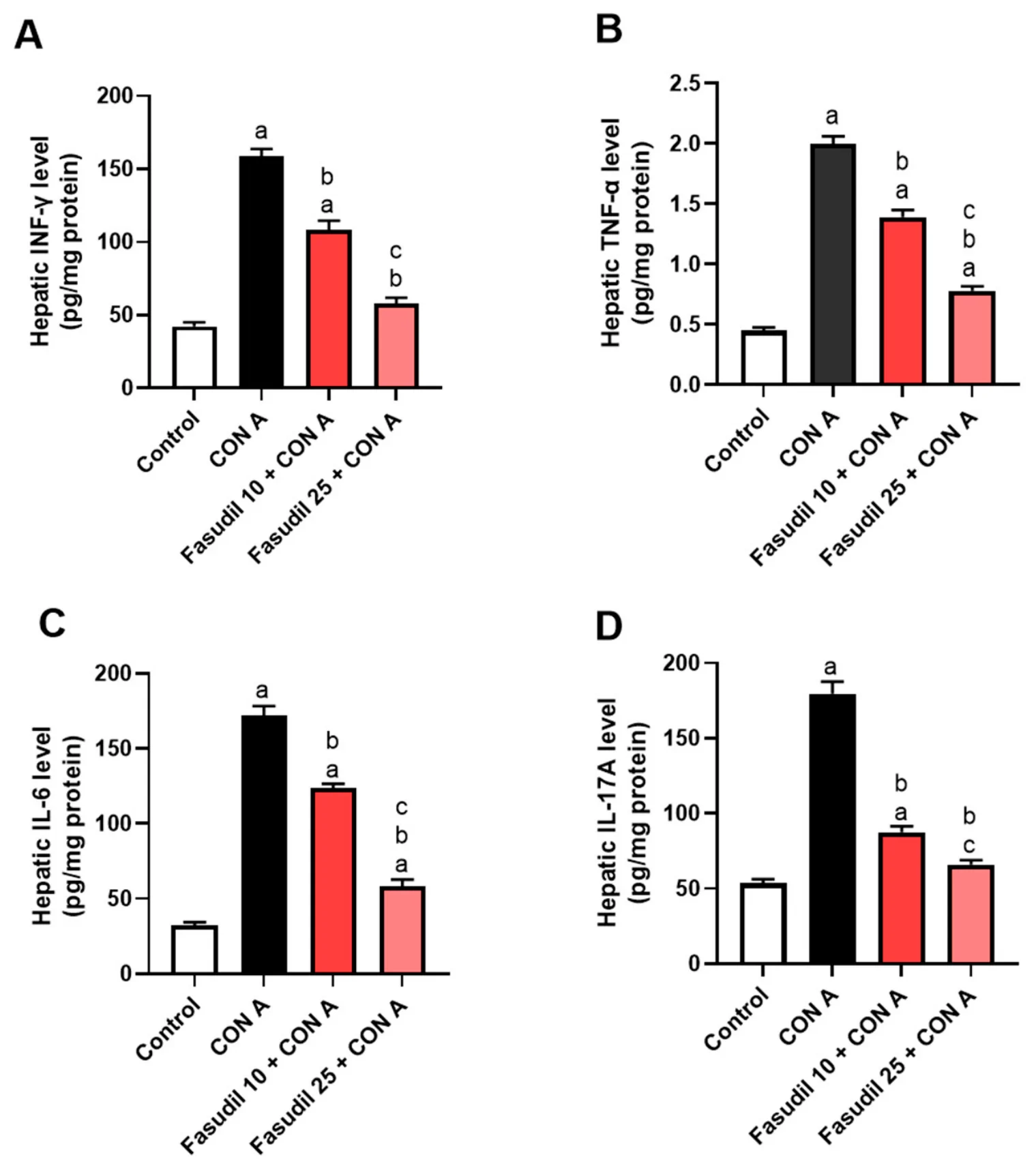
Fasudil attenuates concanavalin A (Con A)-induced elevations of hepatic pro-inflammatory cytokines (INF-γ, TNF-α, IL-6, IL-17A). (**A**) Interferon gamma (INF-γ), (**B**) tumor necrosis factor alpha (TNF-α), (**C**) interleukin 6 (IL-6), and (**D**) interleukin 17A (IL-17A) (obtained by ELISA). These measurements were performed using liver tissue homogenates. Data are expressed as mean ± SEM, *n* = 4. One-way ANOVA followed by Tukey–Kramer multiple comparisons post hoc test. *p* ≤ 0.05 was considered statistically significant. ^a,b,c^, *p* ≤ 0.05 was considered significantly different relative to control, Con A, or Fasudil 10 + Con A groups, respectively.

The oxidative stress and the activation of CD4^+^ and CD8^+^ T cells stimulate the release of pro-inflammatory cytokines, including TNF-α, IL-6, IL-17A, and IFNγ. These cytokines make tissue damage worse by bringing monocytes and neutrophils to the site of inflammation and creating a positive feedback loop in which lymphocyte activation leads to the recruitment of innate immune cells, which in turn further activates lymphocytes [35]. To study the protective effect of fasudil on these inflammatory cytokines in AIH, we assessed its levels using the ELIZA technique in hepatic homogenates, as shown in Figure 6. The Con A group significantly increased hepatic levels of INF-γ, TNF-α, IL-6, and IL-17A (Figure 6A–D) compared to the control group. Pretreatment with fasudil (10 and 25 mg/kg) significantly lowered the aforementioned parameters compared to the Con A group. These results show that fasudil protected against Con A-induced liver inflammation, with the fasudil (25 mg/kg) dose having a more marked hepatoprotective effect than the lower dose (10 mg/kg).

### 2.7. Effect of Fasudil on TLR4, NF-κB, NLRP3, Caspase1, and IL-1β Induced by Con A

**Figure 7:**
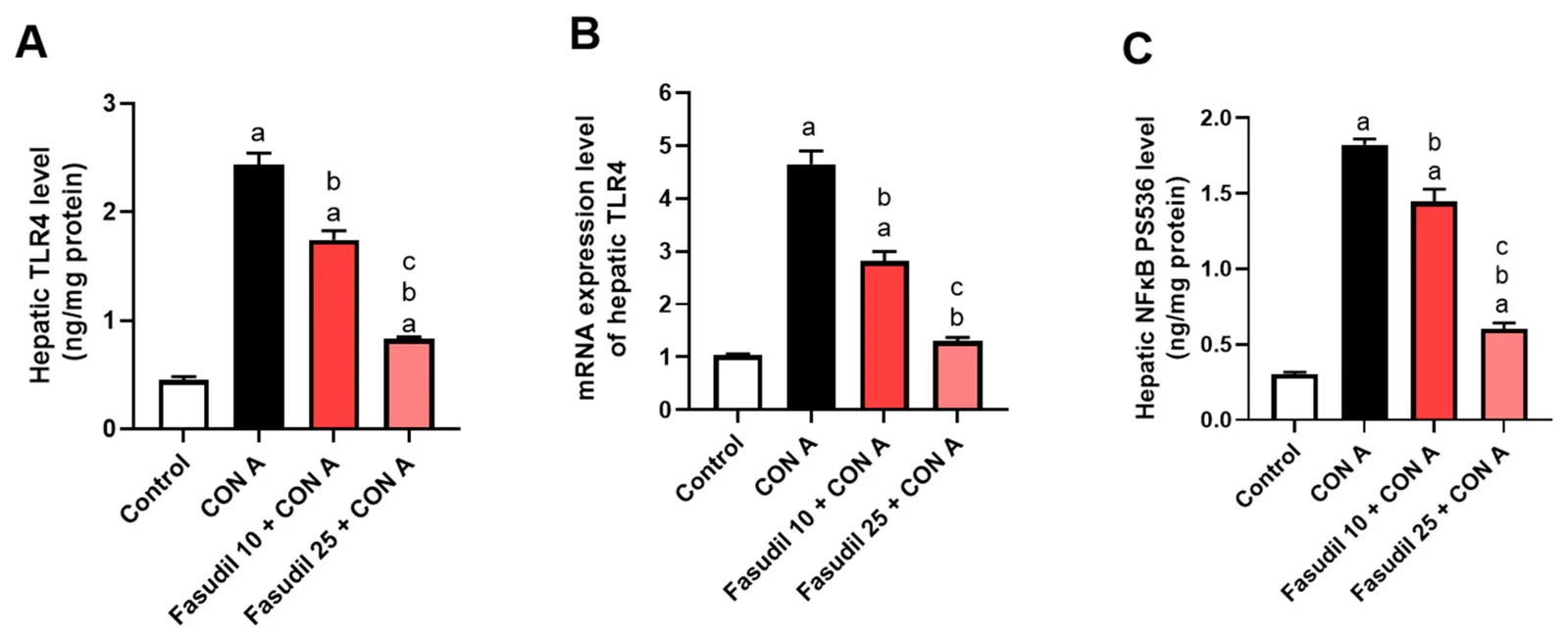
Fasudil mitigates concanavalin A (Con A)-triggered upregulation of hepatic TLR4 and NF-κB: (**A**) Toll-Like Receptor 4 (TLR4) protein levels (obtained by ELISA). (**B**) Toll-Like Receptor 4 (TLR4) mRNA (obtained by PCR). (**C**) Hepatic Nuclear Factor kappa B (NFκB) (obtained by ELISA). These measurements were performed using liver tissue homogenates. Data are expressed as mean ± SEM, *n* = 5. One-way ANOVA followed by Tukey–Kramer multiple comparisons post hoc test. *p* ≤ 0.05 was considered statistically significant. ^a,b,c^, *p* ≤ 0.05 was considered significantly different relative to the control, Con A, or Fasudil 10 + Con A groups, respectively.

**Figure 8:**
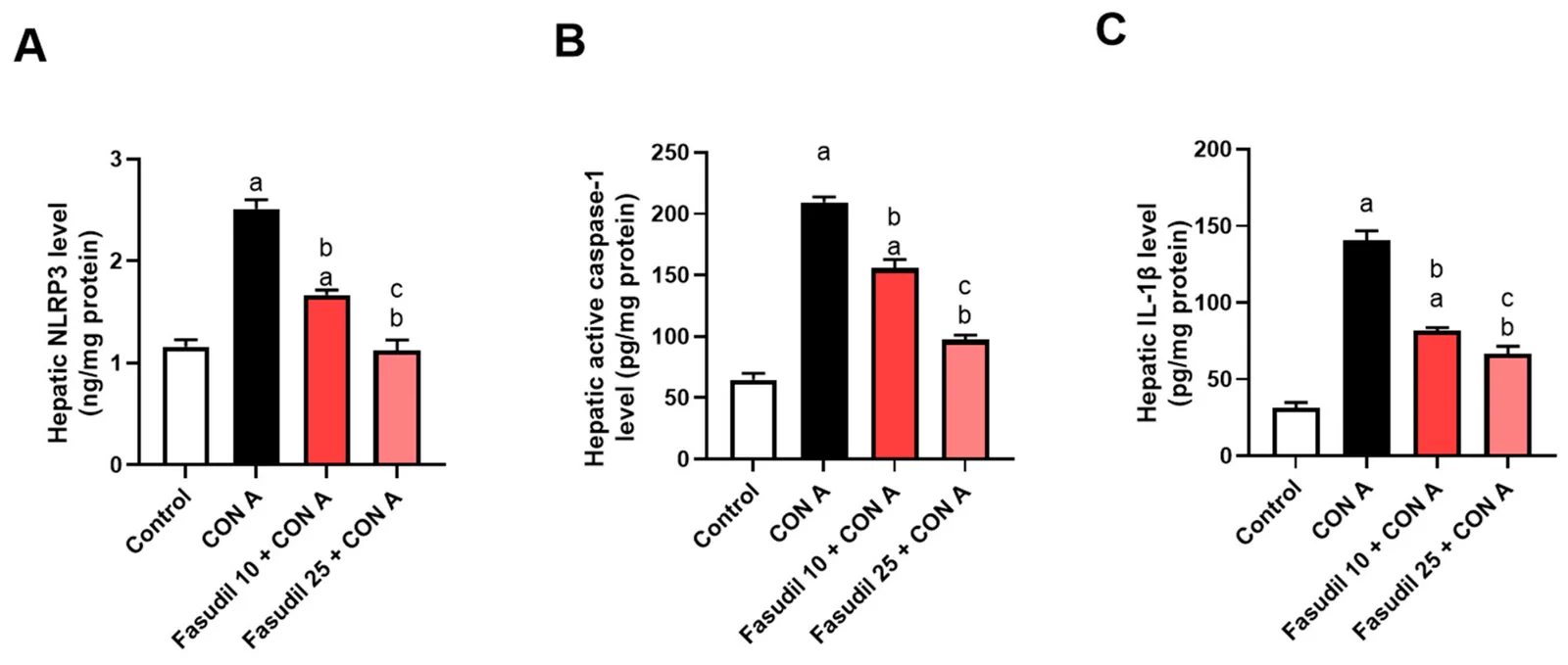
Fasudil mitigates concanavalin A (Con A)-triggered upregulation of NLRP3, inflammasome pathway, and iNOS levels: (**A**) NOD-like receptor family, pyrin domain-containing protein 3 (NLRP3), (**B**) caspase-1, and (**C**) Interleukin-1 Beta (IL-1β) (obtained by ELISA). These measurements were performed using liver tissue homogenates. Data are expressed as mean ± SEM, *n* = 4. One-way ANOVA followed by Tukey–Kramer multiple comparisons post hoc test. *p* ≤ 0.05 was considered statistically significant. ^a,b,c^, *p* ≤ 0.05 was considered significantly different relative to the control, Con A, or Fasudil 10 + Con A groups, respectively.

A key upstream pathway that bridges these innate and adaptive immune responses in AIH is the TLR4/NF-κB signaling cascade [36]. TLR4 serves as a key receptor for sensing inflammatory signals of resident liver macrophages and neutrophils [37]. Its activation in Kupffer cells and neutrophils leads to NF-κB activation with the subsequent production of pro-inflammatory cytokines and activation of inflammosomes, which amplify immune cell recruitment and liver injury. Accordingly, the TLR4/NF-κB signaling pathway in Kupffer cells acts as a critical amplifier of inflammation [38]. We assessed the effect of fasudil on this pathway, as shown in Figure 7 and Figure 8. The Con A group significantly increased hepatic TLR4 levels (Figure 7A), hepatic TLR4 mRNA expression levels (Figure 7B), NF-κB pS536 levels (Figure 7C), NLRP3 (Figure 8A), caspase1 (Figure 8B), and IL-1β (Figure 8C) compared to the control group. Pretreatment with Fasudil (10 and 25 mg/kg) significantly lowered the aforementioned parameters compared to the Con A group. This finding indicates that fasudil provided protection against Con A-induced liver inflammation, with fasudil (25 mg/kg) having a significantly greater hepatoprotective effect than the lower dose (10 mg/kg).

### 2.8. Fasudil Shifts M1 to M2 Polarization via Regulation of iNOS and CD163 Expression

**Figure 9:**
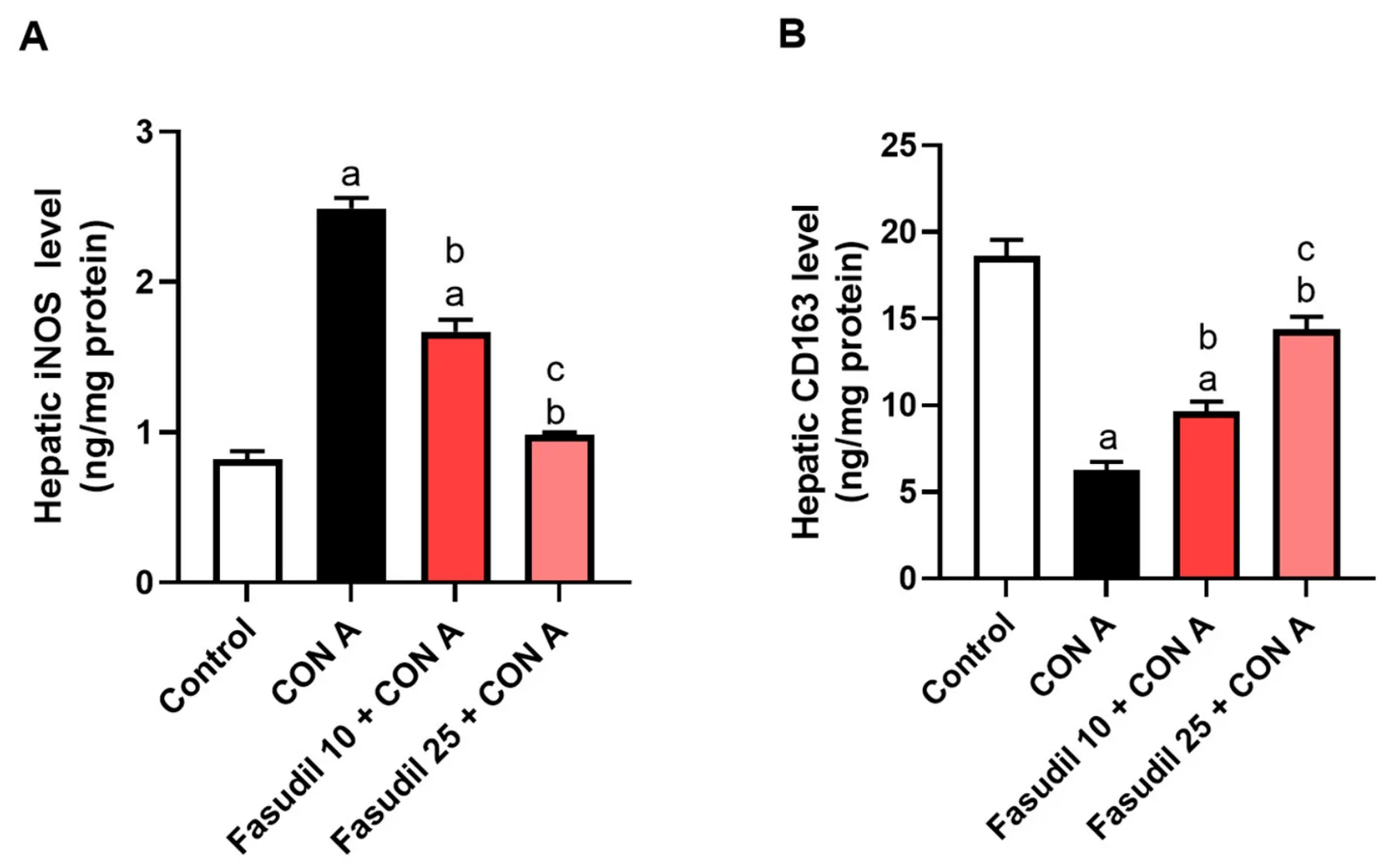
Fasudil modulates hepatic M1/M2 polarization (downregulates iNOS and upregulates CD163 levels): (**A**) Inducible Nitric Oxide Synthase (iNOS); (**B**) Cluster of Differentiation (CD)-163 (obtained by ELISA). These measurements were performed using liver tissue homogenates. Data are expressed as mean ± SEM, *n* = 4. One-way ANOVA followed by Tukey–Kramer multiple comparisons post hoc test. *p* ≤ 0.05 was considered statistically significant. ^a,b,c^, *p* ≤ 0.05 was considered significantly different relative to the control, Con A, or Fasudil 10 + Con A groups, respectively.

To further characterize the changes in macrophage polarization, we assessed the M1 marker iNOS, as well as the M2 marker CD163, using the ELISA technique. The hepatic levels of iNOS were remarkably increased in the Con A group compared to the control group. Fasudil dose-dependently reduced these elevations (Figure 9A). These results indicate that fasudil mediates macrophage polarization through the suppression of M1-related pro-inflammatory markers. CD163, indicative of alternatively activated M2 macrophages that dampen inflammation and support tissue regeneration, was significantly decreased after Con A treatment compared the control group. Notably, fasudil pretreatment succeeded in restoring CD163 levels (Figure 9B). These findings indicate that fasudil-driven M2 macrophage polarization facilitates the resolution of hepatic inflammation and supports tissue repair.

### 2.9. Effect of Fasudil on BCL-2, BAX, and Caspase-3 Induced by Con A

**Figure 10:**
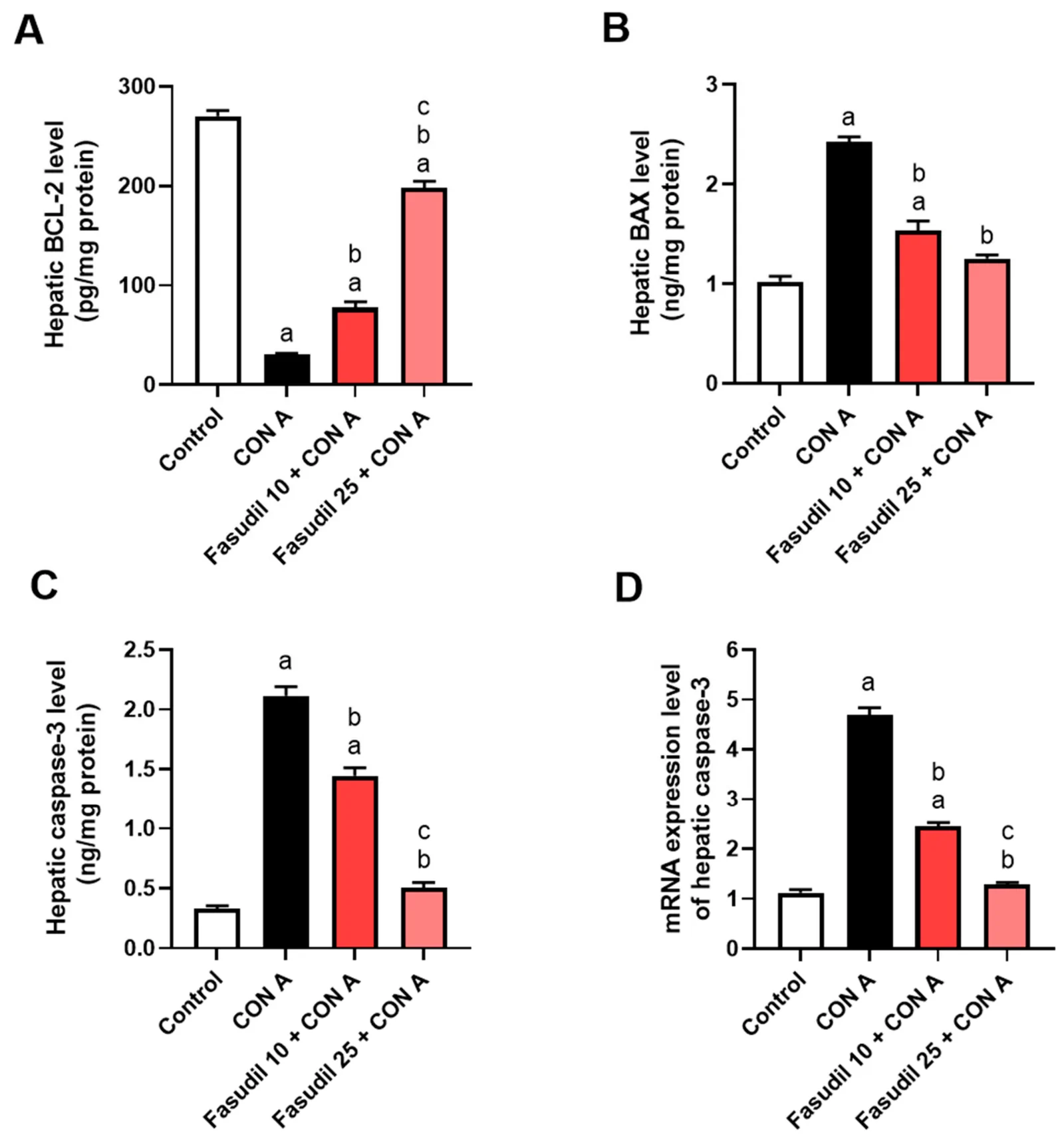
Fasudil restores BCL-2/BAX balance and reduces caspase-3 in concanavalin A (Con A)-induced hepatic apoptosis: (**A**) B cell lymphoma 2 (BCL-2), (**B**) BCL-2-associated X protein (BAX), (**C**) caspase-3 protein levels (obtained by ELISA), and (**D**) Caspase 3 mRNA levels (obtained by PCR). These measurements were performed using liver tissue homogenates. Data are expressed as mean ± SEM, *n* = 4. One-way ANOVA followed by Tukey–Kramer multiple comparisons post hoc test. *p* ≤ 0.05 was considered statistically significant. ^a,b,c^, *p* ≤ 0.05 was considered significantly different relative to the control, Con A, or Fasudil 10 + Con A groups, respectively.

Apoptosis was reported to represent a key mechanism of hepatocellular injury in Con A-induced AIH [39]. As shown in Figure 10A,B, Con A administration significantly lowered hepatic BCL-2 levels while elevating Bax expression, leading to a marked elevation in the Bax/BCL-2 ratio. In parallel, Con A significantly upregulated caspase-3 levels (Figure 10C) and caspase-3 mRNA (Figure 10D) compared to the control group. Pretreatment with fasudil (10 and 25 mg/kg) restored BCL-2 expression, reduced Bax levels, and consequently lowered the Bax/BCL-2 ratio. Fasudil also attenuated caspase-3 activation. Notably, the higher dose (25 mg/kg) exerted a more pronounced protective effect than the lower dose (10 mg/kg).

## 3. Discussion

AIH is a chronic, progressive inflammatory disease of the liver parenchyma mediated by an aberrant autoimmune response. It is characterized by elevated serum transaminase levels and interface hepatitis on histological examination [10,40]. Con A is widely used as an experimental model to investigate acute liver injury, as its underlying mechanisms and histopathological features closely resemble those observed in AIH [23,41]. According to current clinical guidelines, corticosteroids, alone or in combination with azathioprine constitute the first-line therapy for AIH [4,5]. Current clinical primarily guidelines rely on immunosuppressive drugs for AIH. Although they are effective in many patients, they have some limitations [6], which highlights the urgent need to investigate adjunctive or alternative therapeutic strategies. The present study aimed to explore the hepatoprotective effect of fasudil against Con A-induced AIH, giving an insight into its therapeutic potential. Fasudil was administered systemically in the current study, and previous preclinical and clinical studies have reported that it has a favorable safety and tolerability profile. Interestingly, fasudil has already been approved in Japan for cerebral vasospasm management following subarachnoid hemorrhage and has been used clinically with an acceptable safety profile [42]. Accordingly, large post-marketing surveillance studies and a meta-analysis further demonstrated that fasudil is generally well tolerated [43,44,45]. In addition, numerous preclinical studies have shown that systemic fasudil exerts protective effects in inflammation in different organs primarily through inhibition of the ROCK-mediated inflammatory and fibrotic pathways [46,47]. Collectively, our findings, together with the established clinical safety profile of fasudil, provide a rationale for its further preclinical and clinical development as a therapeutic agent for AIH.

Con A injection resulted in severe hepatocellular injury, as shown by significant elevations in serum levels of ALT, AST, ALP, GGT, and LDH, as well as a marked reduction in serum albumin. Although serum albumin is commonly regarded as a marker of chronic liver dysfunction because of its long half-life, acute inflammatory conditions such as Con A-induced hepatitis can rapidly reduce circulating albumin levels through cytokine-mediated suppression of hepatic albumin synthesis, increased vascular permeability, and redistribution into the extravascular compartment. Moreover, Con A injection showed prominent liver injury, accompanied with infiltration of inflammatory cells. Furthermore, the immunohistochemical evaluation showed that Con A significantly stimulated the infiltration of CD4^+^ and CD8^+^ T cells, outlining its role in triggering adaptive immune responses. Consistently, the Con A group exhibited significant elevated levels of key pro-inflammatory cytokines, including IFNγ, TNF-α, and IL-6. As a potent T cell mitogen, Con A activates both CD4^+^ and CD8^+^ cells through T cell receptor cross-linking, resulting in robust cytokine production and cytotoxic responses that initiate and amplify autoimmune liver injury [48]. In parallel, Con A resulted in a dysregulation of the hepatic oxidant–antioxidant balance, as evidenced by increased levels of MDA and alterations in antioxidant defense parameters. Collectively, these results confirm a coherent pathological cascade that involve immune stimulation, oxidative stress, and inflammatory signaling, contributing to the development and progression of AIH [49,50].

The migration of T cells across the endothelium was reported to be highly dependent on the RhoA/ROCK signaling pathway, as efficient transmigration requires coordinated contraction of the T cell uropod during cell movement [32]. ROCK activity has also been shown to be enriched within filamentous and tubular pseudopodia of migrating T cells, where it regulates key processes such as adhesion, migration, differentiation, proliferation, and survival. Increases in ROCK activation have been consistently observed in T cells in autoimmune diseases mouse models [33,34]. A recent study utilizing naive murine T cells has shown that ROCK2 is selectively activated under Th17 conditions, and T cells from heterozygous ROCK2-deficient mice exhibit impaired Th17 differentiation, accompanied by a reduced expression of transcription factors and decreased production of Th17 cytokines [51]. Furthermore, overexpression of ROCK2 in Th17 cells was reported to promote the secretion of inflammatory cytokines. ROCK2 was reported to increase the binding rate of IL-17 and IL-21 promoters, stimulate T cells to secrete IL-10, induce T cells to polarize to Th17/Th1, and inhibit Th2/Treg differentiation [52]. RhoA deficiency in T cells was also reported to impair Th2 differentiation [53]. Collectively, these studies demonstrate a direct crosstalk between RhoA/ROCK2 signaling and lymphocyte activation, proliferation, and differentiation. Although fasudil inhibits both ROCK1 and ROCK2, the present study focuses on ROCK2 because of its well-established role in immune regulation and inflammatory signaling. Consistent with these findings, our study shows that inhibition of the RhoA/ROCK2 pathway by fasudil is associated with reduced activation of both CD4^+^ T cells and CD8^+^ T in AIH, further supporting the critical role of ROCK signaling in regulating T cell-mediated immune responses.

T lymphocytes play a central role in the pathogenesis of AIH, and Con A shows a robust activation of these cells with subsequent hepatocyte apoptosis and dysregulated cytokine production, which constitute the key pathological features in AIH [54]. The immunohistochemical examination in our study aligns with this cascade, showing a significant expression of CD4^+^ T cells and CD8^+^ T cells in the Con A group. Notably, pretreatment with fasudil significantly attenuated these changes, which can be explained by its inhibitory effect on the RhoA/ROCK2 pathway. Moreover, our results demonstrated that Con A markedly induced the expression of INF-γ and IL-17A, which was significantly attenuated by fasudil-associated inhibition of RhoA/ROCK2, indicating not only its effect on CD4^+^ T cell infiltration, as observed by immunohistochemistry, but also its modulatory role in CD4^+^ T cell differentiation.

Another deriver of Con A-induced AIH is oxidative stress [28], which alters gene expression and promotes apoptosis, necrosis, and fibrosis [29]. Our study shows that Con A significantly increased hepatic MDA while reducing antioxidant enzymes, as shown by the TAC, SOD, and GSH levels. The RhoA/ROCK pathway has been reported to play a role in oxidative stress in multiple pathological conditions [30,31]. Consistent with previous reports showing the antioxidant effects of fasudil [55], our findings demonstrate that fasudil effectively reversed Con A-induced oxidative stress by restoring antioxidant defenses and decreasing lipid peroxidation, further supporting its hepatoprotective potential in AIH.

Oxidative stress and the activation of CD4^+^ and CD8^+^ T cells stimulate the release of pro-inflammatory cytokines, including TNF-α, IL-6, IL-17A, and IFNγ. These cytokines make tissue damage worse by bringing monocytes and neutrophils to the site of inflammation, and they create a positive feedback loop in which lymphocyte activation leads to the recruitment of innate immune cells, which in turn further activates lymphocytes [35]. The activation of CD4^+^ T cells, which serve as major producers of IFNγ, and their differentiation into Th17 cells, which are major producers of the pro-inflammatory cytokine IL-17A, promotes the recruitment of monocytes and neutrophils to the site of inflammation, further amplifying hepatic immune responses [56].

A key upstream pathway that bridges this innate and adaptive immune responses in AIH is the TLR4/NF-κB signaling cascade [36]. Kupffer cells, the liver’s resident macrophages, play a central role in hepatic innate immunity, with TLR4 serving as a key receptor for sensing inflammatory signals. TLR4 also serves as a key mediator of neutrophil-driven inflammatory responses [37]. TLR4 activation in Kupffer cells and neutrophils leads to NF-κB activation and the production of pro-inflammatory cytokines such as TNF-α and IL-6, which amplify immune cell recruitment and liver injury. Accordingly, the TLR4/NF-κB signaling pathway in Kupffer cells acts as a critical amplifier of inflammation [38].

The infiltration of immune cells is a central driver in the development and progression of liver injury. These infiltrating cells not only release pro-inflammatory mediators, but also recruit additional leukocytes into hepatic tissue, which amplify and sustain the inflammatory cascade [57]. Among the earliest responders in Con A-induced AIH are neutrophils [58]. Neutrophils play a central role in the evolving immune response by recruiting other leukocytes such as macrophages [59]. Consistently, the histopathological examination in our study showed prominent inflammatory cell infiltration and evidenced Kupffer cell activation. Our study shows that fasudil-associated inhibition of RhoA/ROCK pathway was accompanied with a downregulation of TLR4/NF-κB signaling cascade, which resulted in decreased macrophage infiltration and iNOS expression with a subsequent decrease in inflammatory cytokines. Consistently, fasudil was found to suppress TLR4/NF-κB signaling in EAE models with a subsequent reduction in the production of inflammatory cytokines [47]. Furthermore, fasudil was reported to attenuate liver injury via inhibiting NF-κB [60]. Our findings align with these studies, showing that fasudil significantly decreased Con A-induced RhoA/ROCK stimulation, which was accompanied by a downregulation in TLR4/NF-κB signaling pathway. This was accompanied by a significant decrease in downstream inflammatory cytokines, including IL-6, IL-17A, INF-γ, and TNF-α.

Macrophage polarization is reported as a critical regulator of AIH progression, with classically activated M1 macrophages amplifying hepatic inflammation through the production of pro-inflammatory mediators, whereas alternatively activated M2 macrophages promote the resolution of inflammation and tissue repair [61]. In the present study, fasudil suppressed the M1-associated marker iNOS while restoring the M2-associated marker CD163, suggesting that ROCK inhibition shifts macrophage polarization from a pro-inflammatory to a pro-resolving phenotype. This phenotypic switch is associated with reduced production of inflammatory mediators, enhanced resolution of inflammation, and improved tissue repair, which may contribute to the hepatoprotective effects of fasudil observed in the present study. Our findings are also in agreement with previous reports showing that fasudil attenuates inflammatory responses by modulating macrophage polarization [62].

The inhibitory effect of fasudil on RhoA/ROCK2 signaling, together with the downregulation of the TLR4/NF-κB pathway, likely played a critical role in suppressing both the priming and activation of the NLRP3 inflammasome. This is because TLR4/NF-κB signaling provides the essential priming signal required for NLRP3 inflammasome assembly, leading to subsequent caspase-1 activation and the maturation of pro-inflammatory cytokines [63].

In parallel, apoptosis was reported to represent a key mechanism of hepatocellular injury in Con A-induced AIH [39]. It was reported that anti-apoptotic proteins such as BCL-2 preserve mitochondrial integrity, whereas pro-apoptotic proteins such as Bax promote mitochondrial outer membrane permeabilization and downstream caspase activation [64]. In our study, Con A disrupted this balance, stimulating apoptotic signaling and leading to increased caspase-3 activation. Interestingly, the pretreatments with fasudil significantly restored the balance and decreased the levels of Bax and caspase-3, as shown by normalization of BCL-2 expression. These findings align with previous studies. For example, Goe et al. reported that fasudil attenuated apoptosis by upregulating BCL-2 and downregulating Bax expression, suggesting its cytoprotective effects [65].

Although the present study demonstrates that fasudil treatment was associated with the suppression of both RhoA/ROCK and TLR4/NF-κB signaling, our findings do not establish the signaling hierarchy between these pathways. In the Con A model, T cell activation is the primary initiating event, whereas activation of the TLR4/NF-κB pathway is believed to amplify hepatic inflammation through activation of Kupffer cells and infiltrating macrophages in response to endogenous danger-associated molecular patterns released from injured hepatocytes [16,17,18,66]. Therefore, the observed reduction in TLR4/NF-κB signaling may reflect either a direct consequence of ROCK inhibition or a secondary effect resulting from attenuation of hepatic injury and inflammation. Future studies employing selective pharmacological inhibitors or genetic approaches will be necessary to determine the precise mechanistic relationship between ROCK and TLR4/NF-κB signaling in autoimmune hepatitis. Nevertheless, previous studies have demonstrated that ROCK knockdown suppresses NF-κB activation [67,68], while selective ROCK2 inhibition attenuates TLR4-mediated inflammatory signaling [68]. Together, these genetic and pharmacological studies support our findings and suggest that ROCK functions upstream of the TLR4/NF-κB signaling pathway.

The novelty of the present study lies in extending the therapeutic application of fasudil-associated inhibition of RhoA/ROCK to autoimmune hepatitis, a predominantly immune-mediated liver disease in which both adaptive and innate immune responses contribute to disease progression. Unlike previous studies of fasudil in toxic or ischemic liver injury, our findings demonstrate that fasudil-associated inhibition of RhoA/ROCK is accompanied by attenuation of CD4^+^ and CD8^+^ T cell infiltration together with suppression of inflammatory signaling, oxidative stress, and apoptosis. However, we recognize some limitations of the present study. Although immunohistochemistry and ELISA analyses of CD markers suggest modulation of immune cell infiltration, these approaches do not conclusively define specific immune cell subsets, polarization states, or the cellular sources of cytokine production. In addition, ELISA measurements of cytokines in liver homogenates reflect overall cytokine levels but do not distinguish cytokine-producing cells. Future studies using intracellular cytokine staining coupled with flow cytometry will provide a more comprehensive characterization of immune cell populations, activation states, and cytokine-producing subsets, further defining the immunomodulatory effects of fasudil. Third, although fasudil treatment was associated with suppression of the RhoA/ROCK, oxidative stress, and TLR4/NF-κB signaling pathways, with a subsequent attenuation of hepatic inflammation, apoptosis, and liver injury, the present study does not establish a direct causal relationship between these molecular changes and its hepatoprotective effects. Future studies using pathway-specific inhibitors or genetic models, such as ROCK knockout mice, will further define the causal role of ROCK signaling in disease pathogenesis.

In conclusion, inhibition of RhoA/ROCK2 pathway by fasudil was associated with reduction in lymphocytes activation and oxidative stress, which was accompanied by downregulation of the TLR4/NF-κB pathway. This effect was associated with reduced production of various pro-inflammatory cytokines and attenuation of macrophage activation. It also restored balance among key regulators of apoptosis. Our results highlight fasudil-associated RhoA/ROCK2 inhibition as a therapeutic potential in AIH management.

## 4. Materials and Methods

### 4.1. Chemicals

Fasudil and Con A were obtained from Maclin (China, Cat. F845491) and Sigma-Aldrich (St. Louis, MO, USA), respectively.

### 4.2. Animals

We obtained the mice used in this study, male BALB-c mice weighing 25–30 g, from the Medical Experimental Research Center (MERC), Egypt. Under standard environmental conditions, they were kept six per cage, with ad libitum access to food and water. We followed the ethical guidelines approved by Mansoura University Animal Care and Use Committee in all experimental procedures of our study (Approval Code: PHARM.MS.24.5.29).

### 4.3. Experimental Design

We randomly allocated 25 mice into 5 experimental groups (*n* = 5 per group):

1. Control group: Mice received normal saline intraperitoneally (i.p.) daily for 7 days.
2. Fasudil 25 control group: Mice received fasudil (25 mg/kg, i.p.) daily for 7 days.
3. Con A group: Mice received i.p. injection as in the control group, followed by Con A (15 mg/kg, i.p.) on day 7 [69].
4. Fasudil 10 + Con A group: Mice received fasudil (10 mg/kg, i.p.) daily for 7 days, followed by Con A (15 mg/kg, i.p.) on day 7 [70].
5. Fasudil 25 + Con A group: Mice received fasudil (25 mg/kg, i.p.) daily for 7 days, followed by Con A (15 mg/kg, i.p.) on day 7 [71].

Fasudil was dissolved in sterile saline (0.9% NaCl) as the vehicle and administered at a final injection volume of 10 mL/kg body weight via intraperitoneal injection. The compound purity was 98% (as provided by the manufacturer). Mice were anesthetized with secobarbital (50 mg/kg, i.p.) (in accordance with the institutional ethical guidelines for animal research) six hours after Con A injection to collect blood samples via retro-orbital venous plexus puncture. The 6 h time point was selected because it is widely recognized as the peak phase of acute liver injury in the Con A-induced hepatitis model, characterized by maximal hepatocellular injury, inflammatory cytokine production, and histopathological changes, making it an appropriate endpoint for evaluating the acute hepatoprotective effects of fasudil. Blood samples were centrifuged at 672× *g* for 15 min to separate serum for liver function markers. Following anesthesia, the mice were then euthanized by cervical dislocation to isolate the liver and split it into 3 parts. The first part was kept in 10% neutral-buffered formalin for histopathological examination. The second part was homogenized in phosphate-buffered saline (PBS) and centrifuged to get the supernatant for oxidative stress and enzyme-linked immunosorbent assay (ELISA) measurements. The third portion was quick-frozen in liquid nitrogen and stored at −80 °C for PCR and Western blotting.

### 4.4. Liver Function Biochemical Assay

Aspartate aminotransferase (AST) and alanine aminotransferase (ALT) were spectrophotometrically determined using kits obtained from AGAPPE (AGAPPE, Kerala, India, Cat. No. 11409003 and 11408003 for ALT and AST, respectively). Alkaline phosphatase (AGAPPE, Kerala, India, Cat. No. 11401009), Albumin (ALB) (SPECTRUM, Cairo, Egypt, Cat. No. 211001), Lactate dehydrogenase (LDH) (Abcam, Cambridge, UK, Cat. No. ab102526), and gamma-glutamyl transferase (GGT) (SWEMED DIAGNOSTICS, Kerala, India, Cat. No. 11416001) were measured according to the manufacturer's instructions.

### 4.5. Determination of Total Protein Content

Total protein content in liver homogenates was quantified using the Bradford method with a Genei protein assay kit (Bangalore, India).

### 4.6. Determination of Oxidative Stress

Malondialdehyde (MDA), total antioxidant capacity (TAC), glutathione (GSH), and superoxide dismutase (SOD) in hepatic tissue homogenates were measured using kits from Biodiagnostics (Giza, Egypt) according to the manufacturer’s instructions.

### 4.7. Histopathological and IHC Examinations

Liver portions were fixed in 10% neutral-buffered formalin and embedded in paraffin. Paraffin blocks were sectioned at 5 μm thickness, deparaffinized in xylene, and rehydrated using graded ethanol to distilled water. For routine histology, sections were stained with hematoxylin and eosin (H&E) and examined in a randomized, blinded fashion under a light microscope (Olympus CH2, Tokyo, Japan). Multiparametric scoring was used in a blinded manner to assess the histopathological changes in hepatic sections of different treatment groups. The assessed parameters are the extent of cellular damage, sinusoidal dilatation, inflammation, and central vein congestion.

For immunohistochemistry (IHC) [72], blocking of a non-specific binding was carried out by 5% normal goat serum for 30 min at room temperature. Primary antibodies were applied overnight at 4 °C: NF-κB p65 (Cell Signaling Technology, Danvers, MA, USA, catalog #8242), TNF-α (Abcam, Cambridge, UK, catalog # ab92324), Bcl-2-associated X protein (BAX) (Abcam, Cambridge, UK, catalog #ab32503), B cell lymphoma 2 (BCL-2) (Cell Signaling Technology, Danvers, MA, USA, catalog #2764), cleaved caspase-3 (Cell Signaling Technology, catalog #9661), CD4, (Cell Signaling Technology, Danvers, MA, USA, catalog # 25229), and CD8 (Cell signaling Technology, Danvers, MA, USA, catalog #98941). After washing, the sections were incubated with the corresponding secondary antibody conjugated with HRP and counterstained with Mayer’s hematoxylin. All sections were examined under light microscopy in a randomized, blinded manner. Following color deconvolution, the positive immunoreactivity area % was analyzed by image analysis software (ImageJ, version 1.5r, NIH, Bethesda, MD, USA). Five non-overlapping high-power fields with magnifications of 100× and 400× were chosen at random and examined for each region. A uniform manually adjusted threshold was set once and applied equally to all photographs within the same experiment. Hepatocytes were included in the regions of interest, but large vessels and artifacts were left out. The pathologist doing quantification was blinded to the experimental groups.

### 4.8. Determination of ELISA Biomarkers

Hepatic levels of cluster of differentiation (CD)-4^+^ (Cat. No. MBS2506108, MyBioSource, San Diego, CA, USA), CD8^+^ (Cat. No. MBS2516027, MyBioSource, San Diego, CA, USA), CD163 (Cat. No. MBS2516344, MyBioSource, San Diego, CA, USA), ROCK (AFGV Scientific, Arlington Heights, IL, USA, Cat. No. EK731562), INF-γ (Elabscience, Houston, TX, USA, Cat. No. E-EL-M0048), IL-6 (Elabscience, Houston, TX, USA, Cat. No. 431301), IL-17A (Elabscience, Houston, TX, USA, Cat. No. E-EL-M0047), TNF-α (BT LAB, Shanghai, China, Cat. No. E0117Mo), toll-like receptor 4 (TLR4) (Novus Biological, Centennial, CO, USA, Cat. No. NBP2-76570), nuclear factor kappa-B phosphorylated at Serine 536 (NF-κB pS536) (Assay Geni, Dublin, Ireland, Cat. No. SBRS1911), RhoA (Antibody-Online, Limerick, PA, USA, Cat. No. ABIN1058128), caspase-3 (Elabscience, Houston, TX, USA, Cat. No. E-EL-M0238), inducible nitric oxide synthase (iNOS) (Novus Biological, Centennial, CO, USA, Cat. No. NBP2-80256), NLRP3 (Biorbyt, Cambridge, UK, Cat. No. Orb782216), BAX (BioVision, Milpitas, CA, USA, Cat. No. E4512-100), caspase-1 (Cat. No. E-EL-M0201, Elabscience, Houston, TX, USA), Interleukin-1 Beta (IL-1β) (Cat. No. MBS175967, MyBioSource, San Diego, CA, USA) and BCL-2 (Novus Biological, Centennial, CO, USA, Cat. No. NBP2-69946) were measured using commercial ELISA kits according to the manufacturer’s instructions. All ELISA results were normalized to the total protein concentration.

### 4.9. Polymerase Chain Reaction (PCR)

Total RNA was extracted from tissue samples using the Direct-zol RNA Miniprep Plus kit (Cat. No. R2072, Zymo Research Corp., Irvine, CA, USA). The assessment of RNA concentration was done spectrophotometrically at 260 nm. Using gene-specific primers, we performed reverse transcription and PCR amplification in a single step. A StepOne Real-Time PCR System (Applied Biosystems, Foster City, CA, USA) was used under optimized thermal cycling conditions to carry out the reactions. Each target gene’s relative quantification (RQ) was measured and normalized to beta-actin (β-actin) using the comparative threshold cycle method (delta-delta Ct (ΔΔCt)). The 2^−ΔΔCt^ method was used to calculate the RQ of each gene. The sequences are shown in Table 1.

### 4.10. Western Blotting

The extraction of proteins was carried out according to the manufacturer’s instructions for the TriFast™ kit (PEQLAB Biotechnologie GmbH, Erlangen, Germany; distributed by VWR International GmbH, Darmstadt, Germany). Protein concentration was determined using the Bradford assay with Coomassie Brilliant Blue G-250 and bovine serum albumin (BSA) as the standards. The protein samples were separated using SDS-PAGE gel electrophoresis, subsequently transferred onto a polyvinylidene fluoride (PVDF) membrane, blocked with non-fat dry milk, and incubated with primary antibodies against β-actin (Cell Signaling, Cat. No. 4967) as a loading control and RhoA (Cell Signaling Technology, Cat. No. 2117). After washing, membranes were incubated with the corresponding horseradish peroxidase (HRP)-conjugated secondary antibodies. The chemiluminescent signal, after washing, was developed using a chemiluminescent substrate (Bio-Rad, Hercules, CA, USA) and a CCD camera-based imager. The band intensity of the target proteins was normalized against β-actin (the housekeeping protein).

### 4.11. Statistical Analysis

Data analysis was performed and the figures were generated using GraphPad Prism version 9.0 (GraphPad Software, San Diego, CA, USA). All parametric data were expressed as mean ± standard error of the mean (SEM), employing one-way analysis of variance (ANOVA) followed by Tukey’s post hoc test for pairwise comparisons. The Kruskal–Wallis test was carried out to analyze histopathological scores, followed by post hoc Dunn’s multiple comparison tests. *p* < 0.05 was defined as significant.

## Figures and Tables

**Table 1:** Primer sequences used for gene expression analysis. The table lists the forward and reverse primer sequences for Toll-Like Receptor 4 (TLR4), caspase-3, and β-actin, along with their corresponding gene accession numbers.

	The Forward Sequence	The Reverse Sequence	Number of Gene Accession
TLR4	AGCTTCTCCAATTTTTCAGAACTTC	TGAGAGGTGGTGTAAGCCATGC	NM_021297.1
Caspase-3	TGTCATCTCGCTCTGGTACG	AAATGACCCCTTCATCACCA	NM_009810.3
β-actin	CATTGCTGACAGGATGCAGAAGG	TGCTGGAAGGTGGACAGTGAGG	NM_007393.1

## Data Availability

The original contributions presented in this study are included in the article. Further inquiries can be directed to the corresponding author.
